# Preliminary Findings on School Refusal Outcomes in Children and Adolescents Following 1‐Year Psychiatric Outpatient Treatment

**DOI:** 10.1111/eip.70156

**Published:** 2026-03-10

**Authors:** Yoshinori Sasaki, Masahide Usami, Yuki Hakosima, Kumi Inazaki, Yuki Mizumoto, Mitsuhiro Miyamae, Masaya Ito, Katsunaka Mikami, Noa Tsujii, Takayuki Okada, Hidehiko Takahashi

**Affiliations:** ^1^ Department of Psychiatry and Behavioural Sciences Institute of Science Tokyo, Graduate School of Medical and Dental Sciences Tokyo Japan; ^2^ Department of Child and Adolescent Psychiatry National Kohnodai Medical Centre, Japan Institute for Health Security Chiba Japan; ^3^ National Centre for Cognitive Behaviour Therapy and Research National Centre of Neurology and Psychiatry Kodaira Japan; ^4^ Department of Psychiatry Tokai University School of Medicine Kanagawa Japan; ^5^ Department of Child Mental Health and Development Toyama University Hospital Toyama Japan; ^6^ Institute of Biomedical Engineering Institute of Science Tokyo, Centre for Brain Integration Research Tokyo Japan

**Keywords:** adolescent psychiatry, child psychiatry, early intervention, prognostic factors, school refusal, treatment outcomes

## Abstract

**Introduction:**

School refusal affects many children and adolescents receiving psychiatric care; however, predictors of new‐onset refusal and successful return during treatment remain unclear. This retrospective cohort study identified factors associated with (1) developing school refusal among initially attending patients and (2) returning to school among those initially refusing.

**Methods:**

We reviewed 235 psychiatric outpatients younger than 15 years who continued treatment for 1 year after a first visit between April 2022 and March 2023. Patients were categorised into maintained attendance (*n* = 131), developed school refusal (*n* = 12), resumed attendance (*n* = 34) and persistent refusal (*n* = 58). Demographic variables, diagnoses, prior absence duration and psychiatrist experience were analysed using chi‐squared tests, Fisher's exact tests, Mann–Whitney U tests and multivariate logistic regression.

**Results:**

Among 143 patients attending school at baseline, 12 (8.4%) developed school refusal during treatment; these patients were older (11.8 ± 2.0 vs. 9.2 ± 3.0 years, *p* < 0.01) and more likely to have divorced/separated parents (33.3% vs. 6.9%, *p* < 0.05) than were those who maintained attendance. Among 92 patients already refusing school, 34 (37.0%) resumed attendance. In multivariate analysis, a refusal duration < 1 year independently predicted return to school (odds ratio: 3.28, 95% confidence interval: 1.32–8.15, *p* < 0.05).

**Conclusions:**

Shorter refusal duration was associated with a higher likelihood of successful return to school, underscoring the importance of early intervention. Older children and those from separated families were more vulnerable to developing school refusal, supporting the need for targeted preventive strategies.

## 1 Introduction

School refusal has emerged as a significant public health concern, affecting children and adolescents worldwide, and is closely linked to mental health disorders. School refusal affects approximately 1%–15% of the school‐age population, with systematic reviews suggesting rates around 5% globally (Heyne et al. 2001; Ulaş et al. 2024). This condition extends beyond an educational issue, representing a complex manifestation of underlying psychiatric disorders. Studies consistently demonstrate strong associations between school refusal and anxiety disorders, depression and other emotional disturbances (Tekin and Aydın 2022). The chronic nature of this problem is particularly concerning, as prolonged absenteeism results in substantial consequences across multiple domains, including increased rates of high school dropout, unemployment, substance misuse and delinquency, while also predicting adverse health outcomes and a higher likelihood of criminal justice involvement (Heyne et al. 2001). The coronavirus disease 2019 pandemic exacerbated this crisis, underscoring the urgent need to identify factors influencing school refusal trajectories to develop effective early intervention strategies (Chen et al. 2024; Fujita et al. 2022).

Children who refuse school frequently present with high rates of psychiatric comorbidity, most notably anxiety and depressive disorders (Biswas and Sahoo 2023; Di Vincenzo et al. 2024). Recent community‐based research has reported that over 80% of school‐refusing youth meet criteria for at least one psychiatric disorder, with elevated separation anxiety and depressive symptoms dominating the clinical profile. Clinic‐based studies have consistently demonstrated that psychiatric comorbidity rates range from 53% to 78% among school‐refusing children and adolescents, with anxiety disorders present in 33%–43% of cases and depressive symptoms in approximately 32.4% of patients (Chen et al. 2024; Di Vincenzo et al. 2024). Furthermore, recent research has expanded perspectives on school refusal, suggesting that—in addition to psychiatric disorders and clinical variables—digital engagement such as internet and social media use may play a role (Osmanlı et al. 2025). In inpatient samples, pronounced social withdrawal and emotional dysregulation further distinguish these patients from peers. Family dynamics also play a pivotal role, as parental distress and dysfunctional home environments strongly predict both onset and persistence of school avoidance. Family separation has been reported in 56.3% of cases, maternal psychiatric illness in 45.1% and paternal psychiatric illness in 28.2% of affected families (Chockalingam et al. 2023; Di Vincenzo et al. 2024). However, key predictors and trajectories of improvement or deterioration remain unclear.

Treatment outcomes for school refusal demonstrate substantial heterogeneity, with early intervention representing the most critical determinant of therapeutic success (Johnsen et al. 2024; Strömbeck et al. 2021). Multi‐modal cognitive‐behavioural interventions, incorporating exposure techniques, skills training and family engagement, have shown promise in randomised controlled trials (Johnsen et al. 2024; Pina et al. 2009). However, treatment response has remained highly variable, with attendance rates improving from 6.2% to only 30.3% at 6‐month follow‐up even in specialised programmes (Strömbeck et al. 2021). Treatment responsiveness shows strong temporal dependency, with absence duration serving as a critical prognostic indicator, although the specific mechanisms underlying this relationship remain unclear. Furthermore, longitudinal trajectory studies have demonstrated that 27% of cases maintain total school absence at follow‐up, with trajectories varying significantly by age and clinical presentation (Benoit et al. 2024). Although demographic, clinical and family factors influence outcomes, systematic identification of specific predictors of improvement and deterioration has remained limited. Previous studies have primarily focused on treatment efficacy rather than prognostic factors, and only a few have examined both positive and negative outcomes within the same cohort (Benoit et al. 2024; Pina et al. 2009). Identifying patients who are most likely to improve or deteriorate during treatment remains essential for guiding clinical decision‐making, optimising resource allocation and enabling early recognition of high‐risk cases requiring intensive intervention.

This study aimed to identify predictors of both improvement and deterioration in school refusal over 1 year of continuous child and adolescent psychiatric treatment. We evaluated demographic factors such as age, sex and parental marital status; clinical variables including diagnosis and duration of prior school refusal; and treatment characteristics such as clinician experience in relation to four trajectories: maintained attendance, developed refusal, resumed attendance and persistent refusal. We hypothesised that younger age and shorter prior school refusal duration would predict positive attendance outcomes, whereas neurotic and stress‐related disorders and family instability would be associated with deterioration in school attendance.

## 2 Materials and Methods

### 2.1 Study Design and Participants

**FIGURE 1:**
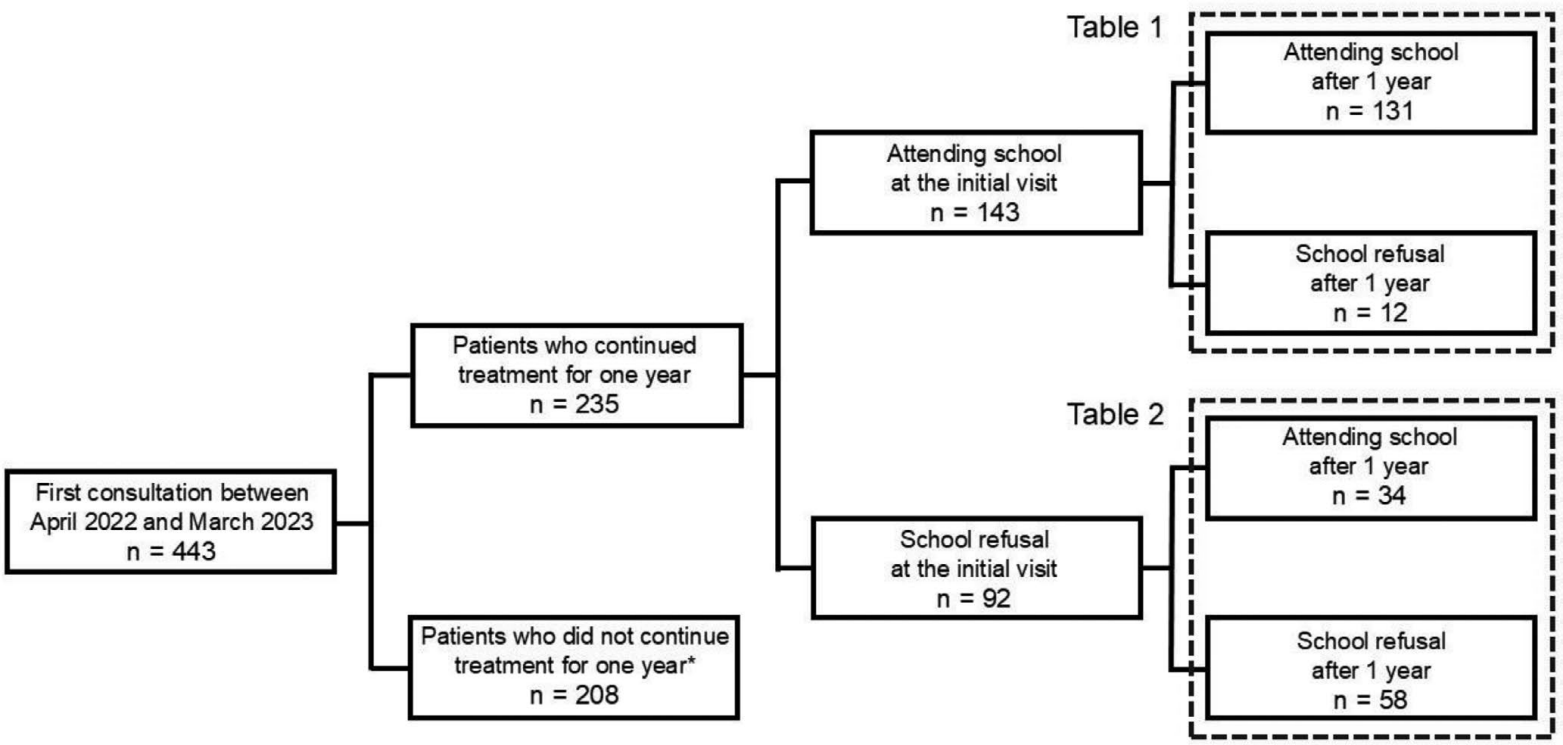
Flowchart showing participant categorisation for Table 1 and Table 2. *This group includes 53 patients who changed their primary psychiatrist, 36 who completed treatment early due to clinical improvement, 74 who discontinued attendance within 1 year, 34 who transferred to another medical institution, and 11 who stopped treatment for other reasons.

This retrospective cohort study examined child and adolescent outpatients who continued treatment for 1 year following their initial visit to the Department of Child and Adolescent Psychiatry, National Kohnodai Medical Centre, Japan. The inclusion criteria were: (A) first visit occurred between 1 April 2022 and 31 March 2023; (B) age under 15 years at the time of the initial visit; and (C) continued treatment for 1 year after their initial visit. Exclusion criteria included moderate‐to‐severe intellectual disability, organic brain disease, drug‐induced psychiatric disorders, traumatic brain injury, or genetic syndromes. During the study period, 443 patients had their first visit to the department. Regarding the inclusion criterion (C), this facility is a training centre for child and adolescent psychiatry where residents and fellows rotate regularly. To ensure consistent longitudinal assessment over the 1‐year period, patients whose primary attending psychiatrist changed during follow‐up were excluded. Specifically, 53 patients experienced a change in attending psychiatrist and could not be followed by the same clinician for the entire year. Additionally, 36 patients completed treatment due to clinical improvement, 74 discontinued outpatient attendance within 1 year, 34 transferred to another medical institution and 11 ended treatment for other reasons. For these patients, as consistent evaluation by the same attending psychiatrist was not possible, regardless of any subsequent symptom changes, they were excluded from the analysis. Among the 235 patients who fulfilled the inclusion and exclusion criteria, 143 were attending school and 92 were not (school refusal) at baseline (Figure 1). No missing data were found for any of the variables included in the analyses.

The center has a specialised training system in child and adolescent psychiatry, where both senior and trainee psychiatrists examine patients. An initial interview was conducted by a psychologist, followed by a comprehensive assessment by a psychiatrist based on the preliminary findings. Patients and their parents were interviewed to collect demographic and clinical information. Patients meeting the exclusion criteria were generally referred to other institutions before initial consultation.

This study was approved by the Institutional Review Board of the Japan Institute for Health Security (NCGM‐G‐003042‐08, NCGM‐S‐004487‐01, NCGM‐S‐004843‐00; Tokyo, Japan) and adhered to the principles of the Declaration of Helsinki and its later amendments. According to the Ethical Guidelines for Medical and Health Research Involving Human Participants established by the Ministry of Health, Labour and Welfare of Japan, written informed consent is not required for retrospective studies using existing clinical data that pose minimal risk to participants when an opt‐out process is implemented, and information about the study is made publicly available. Accordingly, the study information was disclosed on the institutional website to allow patients the opportunity to decline participation.

### 2.2 Data Collection and Measures

The following variables were collected for all participants: sex, age, parental divorce or separation, whether the psychiatrist in charge was a senior or trainee, and duration of prior school refusal at the initial visit. Parental divorce or separation was coded based on information obtained during clinical interviews regarding current cohabiting family members and the legal marital status of the parents; this variable was used to describe family structure rather than to infer family functioning or discord. Diagnoses were made according to the International Classification of Diseases, 10th Revision (ICD‐10) (World Health Organization 1992), following clinical interviews by psychiatrists. Diagnoses reflected the conditions that best captured the patients' symptomatology. Each patient's diagnosis and overall condition were reviewed and evaluated at a multidisciplinary conference, including psychiatrists, clinical psychologists and social workers, to improve diagnostic accuracy. In addition to clinical interviews, standardised psychological scales—such as measures of neurodevelopmental traits, anxiety and depression—were utilised to inform diagnostic decision‐making.

In Japan, school refusal is defined as absence from school for ≥ 30 days within an academic year due to psychological, emotional, physical or social factors (Ministry of Education, Culture, Sports, Science, and Technology 2023). School refusal status was assessed at two time points. At the initial visit, the presence or absence of school refusal was determined based on information obtained from interviews with patients and their parents. At the 1‐year follow‐up, school refusal status was evaluated by the treating psychiatrists through clinical assessment, incorporating information gathered during regular psychiatric consultations throughout the treatment. Given the retrospective design, a strict quantitative attendance threshold could not be applied; clinicians assessed improvement based on reports of attendance frequency and underlying reasons for absence elicited during clinical interviews with patients and families.

### 2.3 Outcome Classification and Statistical Analysis

Based on school attendance status at baseline and at the 1‐year follow‐up, participants were classified into four outcome groups reflecting different school attendance trajectories: (1) maintained attendance—participants attending school regularly at baseline and 1‐year follow‐up, without school refusal; (2) developed school refusal (deterioration)—participants attending school regularly at baseline who developed school refusal (persistent absence from school due to psychiatric or psychosocial factors, not illness or economic hardship) during the treatment period; (3) resumed attendance (improvement)—participants exhibiting school refusal at baseline whose attendance improved sufficiently so that school refusal was no longer evident at the 1‐year follow‐up, as determined by clinical assessment during routine outpatient care; and (4) persistent refusal—participants exhibiting school refusal behaviour at baseline and 1‐year follow‐up. School refusal behaviour was clinically determined based on the child and adolescent psychiatrist's assessment of attendance patterns and underlying psychiatric or psychosocial factors elicited through clinical interviews and evaluations of patients and their families. This included consideration of full and partial attendance improvements, such as increased school engagement or reduced absence frequency, reflecting the heterogeneity of improvement in routine clinical practice.

Pearson's chi‐squared and Fisher's exact tests were used to compare categorical variables between groups, whereas the Mann–Whitney U test was used for continuous variables. Given the exploratory nature of these analyses, no corrections were made for multiple comparisons, and *p*‐values should be interpreted as descriptive rather than confirmatory. All tests were two‐tailed, and statistical significance was set at *p* < 0.05. For 2 × 2 comparisons of binary variables, effect sizes were calculated using the Φ coefficient. Multivariate logistic regression analysis was performed only for patients with school refusal at baseline, as the small number of patients who developed school refusal during treatment (*n* = 12) precluded reliable multivariate modelling for this outcome. Based on prior research, clinical experience and statistical considerations, variables such as sex, age and school refusal lasting < 1 year were included in the multivariate logistic regression analysis. Overlapping categories for school refusal duration (‘< 1 year’, ‘< 2 years’, ‘< 3 years’) were presented descriptively to illustrate prognostic differences, but only the binary classification (‘< 1 year’ vs. ‘≥ 1 year’) was entered into regression modelling to reduce multicollinearity and ensure clinical distinction. All statistical analyses were conducted using IBM SPSS Statistics version 27.0 (Chicago, Illinois, USA).

## 3 Results

Overall, 235 child and adolescent psychiatric outpatients who continued treatment for 1 year after their initial visit were included in the analysis. At their first visit, 143 patients were attending school, and 92 exhibited school refusal. After 1 year, 131 of the 143 patients (91.6%) who had initially been attending school‐maintained attendance, whereas 12 (8.4%) developed school refusal. Among the 92 patients with school refusal at baseline, 34 (37.0%) resumed attendance, and 58 (63.0%) persisted in refusal after 1 year (Figure 1). Of the 92 patients who presented with school refusal at the initial visit, 40 (44.4%) had a duration of < 1 year. Among these, 21 individuals (52.5%) showed improvement at 1‐year follow‐up, while 19 (47.5%) remained school refusers.

When comparing patients who deteriorated (*n* = 12) with those who showed no change (*n* = 131) among individuals initially attending school (Table 1), the mean age of the deterioration group was significantly higher than that of the no‐change group (11.83 ± 2.04 vs. 9.20 ± 3.00 years; effect size = 0.25, *p* < 0.01). Parental divorce or separation was also more common in the deterioration group (33.3% vs. 6.9%; Fisher's exact *p* < 0.05). No significant differences were observed in sex distribution or psychiatrist seniority (*p* = 0.17 and *p* = 0.06, respectively). Diagnostic category differed between groups, with neurotic, stress‐related and somatoform disorders (F4) more prevalent in the deterioration group (58.3% vs. 12.2%; Fisher's exact *p* < 0.01) (Table 1). Specifically, within the deterioration group, F4 diagnoses included other anxiety disorders (F41, *n* = 3), phobic anxiety disorders (F40, *n* = 2), reaction to severe stress and adjustment disorders (F43, *n* = 1) and other nonpsychotic mental disorders (F48, *n* = 1).

**TABLE 1:** Comparison of cases of deterioration and no change in patients attending school at the initial visit.

Characteristics							
	Overall (*n* = 143)	Deterioration (*n* = 12)	No change (*n* = 131)	OR	95% CI	Effect size	*p*
Age^a^	9.42 ± 3.02	11.83 ± 2.04	9.20 ± 3.00	—	—	0.25	< 0.01
Sex							
Male	75 (52.4%)	4 (33.3%)	71 (54.2%)	0.42	0.12–1.47	0.12	0.17
Female	68 (47.6%)	8 (66.7%)	60 (45.8%)	—	—	0.12	0.17
Parental divorce or separation	13 (9.1%)	4 (33.3%)	9 (6.9%)	6.78.	1.71–26.89	—^b^	< 0.05
Senior psychiatrist in charge	96 (67.1%)	5 (41.7%)	91 (69.5%)	0.31	0.09–1.05	—^b^	0.06

Diagnostic classification							
	Overall (*n* = 143)	Deterioration (*n* = 12)	No change (*n* = 131)	OR	95% CI	Effect size	*p*
F2	0	0	0	—	—		
F3	3 (2.1%)	0	3 (2.3%)	0.98	0.95–1.00	—^b^	1.00
F4	23 (16.1%)	7 (58.3%)	16 (12.2%)	10.06	2.85–35.52	—^b^	< 0.01
F5	15 (10.5%)	0	15 (11.5%)	0.89	0.83–0.94	—^b^	0.37
F6	2 (1.4%)	0	2 (1.5%)	0.99	0.96–1.00	—^b^	1.00
F7	15 (10.5%)	1 (8.3%)	14 (10.7%)	0.76	0.09–6.34	—^b^	1.00
F8	43 (30.1%)	1 (8.3%)	42 (32.1%)	0.19	0.02–1.54	—^b^	0.11
F9	42 (29.4%)	3 (25.0%)	39 (29.8%)	0.79	0.20–3.06	—^b^	1.00

*Note:* F2 = Schizophrenia, schizotypal and delusional disorders. F3 = Mood disorders. F4 = Neurotic, stress‐related and somatoform disorders. F5 = Behavioural syndromes associated with physiological disturbances and physical factors. F6 = Disorders of adult personality and behaviour. F7 = Intellectual disabilities. F8 = Disorders of psychological development (e.g., autism spectrum disorder). F9 = Behavioural and emotional disorders with onset in childhood and adolescence (e.g., attention‐deficit/hyperactivity disorder).

Abbreviations: CI, confidence interval; OR, odds ratio.

^a^ Mean ± standard deviation.

^b^ Fisher's exact test was applied when the expected frequency was < 5; Pearson's chi‐squared values were not calculated.

Among patients with school refusal at baseline, those who improved (*n* = 34) did not differ significantly from those who showed no change (*n* = 58) in mean age (11.65 ± 2.50 vs. 12.29 ± 1.83 years; effect size = 0.09), sex (*p* = 0.22), parental divorce or separation (*p* = 0.60), or psychiatrist seniority (*p* = 0.63) (Table 2). The proportion with refusal duration < 1 year was significantly higher in the improvement group than in the no‐change group (63.6% vs. 33.3%; effect size = 0.29, *p* < 0.01). No diagnostic categories differed between these groups (Table 2).

**TABLE 2:** Comparison of cases of improvement and no change in patients with school refusal at the initial visit.

Characteristics							
	Overall (*n* = 92)	Improvement (*n* = 34)	No change (*n* = 58)	OR	95% CI	Effect size	*p*
Age^a^	12.05 ± 2.11	11.65 ± 2.50	12.29 ± 1.83	—	—	0.09	0.41
Sex							
Male	41 (44.6%)	18 (52.9%)	23 (39.7%)	1.71	0.73–4.02	0.13	0.22
Female	51 (55.4%)	16 (47.1%)	35 (60.3%)	—	—	0.13	0.22
Parental divorce or separation	20 (21.7%)	6 (17.6%)	14 (24.1%)	0.67	0.23–1.96	0.08	0.60
Senior psychiatrist in charge	65 (70.7%)	23 (67.6%)	42 (72.4%)	0.80	0.32–2.00	0.05	0.63
Age at onset of school refusal^a^	10.94 ± 2.52	10.73 ± 2.73	11.07 ± 2.40	—	—	0.04	0.67
School refusal duration under 1 year	40 (44.4%)	21 (63.6%)	19 (33.3%)	3.50	1.43–8.59	0.29	< 0.01
School refusal duration under 2 years	67 (74.4%)	23 (69.7%)	44 (77.2%)	0.68	0.26–1.79	0.08	0.46
School refusal duration under 3 years	77 (85.6%)	28 (84.8%)	49 (86.0%)	0.91	0.27–3.07	—^b^	1.00

Diagnostic classification							
	Overall (*n* = 92)	Improvement (*n* = 34)	No change (*n* = 58)	OR	95% CI	Effect size	*p*
F2	1 (1.1%)	0	1 (1.7%)	0.98	0.95–1.02	—^b^	1.00
F3	3 (3.3%)	1 (2.9%)	2 (3.4%)	0.85	0.07–9.72	—^b^	1.00
F4	51 (55.4%)	19 (55.9%)	32 (55.2%)	1.03	0.44–2.41	0.00	0.95
F5	4 (4.3%)	2 (5.9%)	2 (3.4%)	1.75	0.24–13.03	—^b^	0.63
F6	2 (2.2%)	0	2 (3.4%)	0.97	0.92–1.01	—^b^	0.53
F7	2 (2.2%)	0	2 (3.4%)	0.97	0.92–1.01	—^b^	0.53
F8	21 (22.8%)	7 (20.6%)	14 (24.1%)	0.82	0.29–2.27	0.04	0.70
F9	8 (8.7%)	5 (14.7%)	3 (5.2%)	3.16	0.71–14.17	—^b^	0.14

*Note:* F2 = Schizophrenia, schizotypal and delusional disorders. F3 = Mood disorders. F4 = Neurotic, stress‐related and somatoform disorders. F5 = Behavioural syndromes associated with physiological disturbances and physical factors. F6 = Disorders of adult personality and behaviour. F7 = Intellectual disabilities. F8 = Disorders of psychological development (e.g., autism spectrum disorder). F9 = Behavioural and emotional disorders with onset in childhood and adolescence (e.g., attention‐deficit/hyperactivity disorder).

Abbreviations: CI, confidence interval; OR, odds ratio.

^a^ Mean ± standard deviation.

^b^ Fisher's exact test was applied when the expected frequency was < 5; Pearson's chi‐squared values were not calculated.

Multivariate logistic regression among patients with baseline school refusal showed that refusal duration < 1 year was independently associated with improvement (odds ratio [OR]: 3.28, 95% confidence interval [CI]: 1.32–8.15, *p* < 0.05), after adjustment for sex and age. Neither male sex (OR: 1.53, 95% CI: 0.61–3.82, *p* = 0.36) nor age (OR: 0.90, 95% CI: 0.72–1.11, *p* = 0.31) predicted improvement (Table 3).

**TABLE 3:** Multivariate logistic regression analysis of factors associated with improvement in patients with school refusal at the initial visit.

Characteristics	OR	95% CI	*p*
Sex (male)	1.53	0.61–3.82	0.36
Age	0.90	0.72–1.11	0.31
School refusal duration under 1 year	3.28	1.32–8.15	< 0.05

*Note:* Each parameter was adjusted for sex, age and school refusal duration of less than 1 year.

Abbreviations: CI, confidence interval; OR, odds ratio.

## 4 Discussion

This study investigated factors associated with deterioration and improvement of school refusal among child and adolescent psychiatric outpatients who remained in treatment for 1 year. Overall, most patients who attended school at baseline continued to do so after 1 year, whereas a minority developed school refusal. These findings are consistent with those of prior longitudinal studies showing that most children attending school at baseline maintain attendance, whereas a significant proportion of those with initial school refusal exhibit persistent absenteeism after 1 year, with improvement rates of 30%–45% (Egger et al. 2003; Heyne et al. 2001).

Among patients who attended school at their initial visit, older age and family disruption emerged as salient risk factors for deterioration. The deterioration subgroup was, on average, > 2 years older than peers who maintained attendance—consistent with recent evidence that adolescent age is independently associated with increased risk for school refusal, possibly due to heightened academic and social complexities encountered during adolescence (Chen et al. 2024). Parental divorce or separation was nearly five times more prevalent, aligning with research that links dysfunctional family processes and elevated parental psychological difficulties to a greater likelihood of school refusal (Chen et al. 2024; Ulaş and Seçer 2024). These findings suggest that adolescents—who typically face greater academic and social pressures—and those experiencing family instability may be particularly vulnerable to emerging school refusal behaviour. Furthermore, neurotic, stress‐related and somatoform disorders (ICD‐10 F4) were also markedly overrepresented in the deterioration group, reinforcing systematic review findings that anxiety and somatic symptoms are among the most common precursors and correlates of school refusal (Li et al. 2021).

Conversely, among patients with baseline school refusal, the only factor independently predicting return to school was shorter refusal duration (< 1 year). Patients with recent onset of refusal were over three times more likely to resume attendance after adjusting for age and sex, consistent with previous longitudinal research demonstrating that shorter absence duration is associated with better prognosis and higher likelihood of returning to school (Bernstein et al. 2001). This relationship is conceptually reminiscent of findings in schizophrenia research, where longer Duration of Untreated Psychosis is associated with poorer outcomes (Marshall et al. 2005). By analogy, we tentatively propose the idea of a ‘Duration of Untreated School Refusal’ as a hypothesis for future research, whereby prolonged refusal might be associated with more persistent difficulties in returning to school; however, this should be regarded as an exploratory concept rather than an established clinical framework (Penttilä et al. 2014). This framework underscores that school refusal, like first‐episode psychosis, may fall within a critical therapeutic window in which early intervention is more likely to be associated with favourable outcomes. These findings highlight the potential clinical importance of early intervention: prolonged school absence tends to entrench avoidance behaviours, whereas timely therapeutic engagement may support successful reintegration. Evidence from rapid return intervention studies demonstrated 77% reintegration rates compared with 0% among untreated controls (Maeda and Heyne 2019), further corroborated by reports that delayed intervention in adolescent school refusal results in substantially poorer treatment outcomes, with nonresponse rates reaching two‐thirds of cases when developmental complexities compound entrenched avoidance patterns (Heyne 2022). Notably, neither age nor sex influenced improvement, and no specific psychiatric category differentiated improvers from non‐improvers, consistent with evidence that demographic factors rarely predict outcome and that psychosocial interventions remain broadly effective across diagnostic subgroups (Pina et al. 2009).

Collectively, these results emphasise two clinical imperatives. First, students currently attending school but presenting with risk factors—such as older age, family disruption, or anxiety‐related disorders—may require targeted psychosocial support to prevent deterioration. Second, for students already refusing school, prompt assessment and intervention within the first year of onset may be important for increasing the likelihood of return. Early identification programmes and rapid deployment of tailored therapies, including family‐based approaches for structurally vulnerable households and anxiety‐focused interventions, may help improve long‐term attendance outcomes.

### 4.1 Study Limitations

This study had some limitations. First, the design focused exclusively on patients who continued psychiatric consultation and treatment for one full year after their initial visit, potentially excluding those who experienced rapid improvement and discontinued treatment early. This approach may have introduced selection bias towards more persistent cases and underestimated overall improvement rates, as early responders who achieved remission within weeks or months were not captured in the analysis. Additionally, the study design did not allow for examination of potential non‐linear relationships between treatment duration and outcomes, such as rapid improvement occurring within shorter follow‐up periods. Moreover, patients with and without school refusal at baseline represent clinically heterogeneous populations with different diagnostic profiles and referral pathways, which limits the generalisability of our findings and reinforces their primarily descriptive, hypothesis‐generating nature. Second, the retrospective and observational design limited the analysis to data recorded in clinical practice, preventing systematic assessment of emerging factors such as internet and social media use (Osmanlı et al. 2025), as well as detailed aspects of treatment intensity and specific therapeutic modalities. Importantly, confounders such as details of psychosocial interventions, pharmacotherapy, comorbidities and socioeconomic status could not be fully assessed, as these data were not systematically recorded in routine clinical practice. Furthermore, ICD‐10 diagnostic categories were analysed at an upper grouping level (e.g., F4), which provides only a coarse approximation of underlying psychopathology and may have obscured more nuanced diagnostic differences. Additionally, this design precluded confirmation of causal relationships between identified factors and outcomes. In Japan, while school refusal is defined by the Ministry of Education, formal criteria for resumed attendance do not exist; therefore, clinical judgement by the treating psychiatrist was used to determine improvement, which may introduce some measurement variability. Third, the study was conducted at a single specialised child and adolescent psychiatric facility, which may limit generalisability to other clinical settings, healthcare systems, or populations, including those who do not seek psychiatric care or who receive treatment in alternative contexts such as school‐based interventions or primary care. Additionally, the sample was restricted to children aged < 15 years, corresponding to the compulsory education period in Japan; this criterion was chosen to focus on children within the primary and junior high school system, who are subject to unique educational policies and referral pathways. A multicentre study design would increase the sample size and enable more robust multivariable analyses, including multinomial regression approaches.

## 5 Conclusion

This study identified distinct factors associated with both the deterioration into school refusal and improvement in school attendance among child and adolescent psychiatric outpatients over a 1‐year follow‐up. Specifically, for students who were attending school at the initial visit, older age, parental divorce or separation and anxiety‐related disorders emerged as significant risk factors predicting subsequent school refusal. Conversely, among those who presented with school refusal at baseline, a shorter refusal duration (< 1 year) was the only factor that showed a significant association with successful return to school. These findings highlight the importance of early recognition of high‐risk students and suggest that timely assessment and intervention may be associated with better outcomes in managing school refusal. Future research should aim to clarify why a shorter refusal duration predicts better return to school and should investigate the use of targeted prevention programmes for older students and those from separated families. Longer, multicentre studies with larger samples are also required to validate these risk factors and uncover additional predictors over time.

## Funding

This work was supported by the NCGM Intramural Research Fund (24A1014) and JSPS KAKENHI (Grant‐in‐Aid for Early‐Career Scientists, Grant Number JP25K19074). The funders had no role in the conceptualisation, design, data collection, analysis, decision to publish or preparation of the manuscript.

## Ethics Statement

This study was approved by the Institutional Review Board of the Japan Institute for Health Security (NCGM‐G‐003042‐08, NCGM‐S‐004487‐01, NCGM‐S‐004843‐00; Tokyo, Japan) and was conducted in accordance with the principles of the Declaration of Helsinki and its later amendments.

## Consent

The authors have nothing to report.

## Conflicts of Interest

Masahide Usami has received honoraria from Otsuka Pharmaceutical Co. Ltd., and Takeda Pharmaceutical Co. Ltd., as well as manuscript fees from Jiho. Katsunaka Mikami has received financial support from Shionogi & Co. Ltd.; honoraria from Shionogi & Co. Ltd., Sumitomo Pharma Co. Ltd., Takeda Pharmaceutical Co. Ltd. and Otsuka Pharmaceutical Co. Ltd.; travel and accommodation expenses from Otsuka Pharmaceutical Co. Ltd.; honoraria and travel and accommodation expenses for a spouse from Pfizer; and consulting fees from Shionogi & Co. Ltd., EA Pharma Co. Ltd., Sumitomo Pharma Co. Ltd. and Otsuka Pharmaceutical Co. Ltd. The authors declare that they have no competing interests that could have influenced this study.

## Data Availability

Data supporting the findings of this study are available from the Ethical Committee of the National Centre for Global Health and Medicine, Japan, upon reasonable request and with appropriate permission (contact: rinrijm@hosp.ncgm.go.jp). Restrictions apply to the availability of these data, which were used under a license for the current study and are not publicly available.
